# Concurrent Validity Evidence for Pressure-Sensing Walkways Measuring Spatiotemporal Features of Gait: A Systematic Review and Meta-Analysis

**DOI:** 10.3390/s24144537

**Published:** 2024-07-13

**Authors:** Ozell Sanders, Bin Wang, Kimberly Kontson

**Affiliations:** 1 Office of Product Evaluation and Quality, Center for Devices and Radiological Health, US Food and Drug Administration, Silver Spring, MD 20993, USA; ozell.sanders@fda.hhs.gov; 2 Office of Clinical Evaluation and Analysis, Center for Devices and Radiological Health, US Food and Drug Administration, Silver Spring, MD 20993, USA; bin.wang@fda.hhs.gov; 3 Office of Science and Engineering Labs, Center for Devices and Radiological Health, US Food and Drug Administration, Silver Spring, MD 20993, USA

**Keywords:** gait, validity, pressure-sensing walkway, motion capture

## Abstract

Technologies that capture and analyze movement patterns for diagnostic or therapeutic purposes are a major locus of innovation in the United States. Several studies have evaluated their measurement properties in different conditions with variable findings. To date, the authors are not aware of any systematic review of studies conducted to assess the concurrent validity of pressure-sensing walkway technologies. The results of such an analysis could establish the body of evidence needed to confidently use these systems as reference or gold-standard systems when validating novel tools or measures. A comprehensive search of electronic databases including MEDLINE, Embase, and Cumulative Index to Nursing and Allied Health Literature (CINAHL) was performed. The initial search yielded 7670 papers. After removing duplicates and applying study inclusion/exclusion criteria, 11 papers were included in the systematic review with 10 included in a meta-analysis. There were 25 spatial and temporal gait parameters extracted from the included studies. The results showed there was not a significant bias for nearly all spatiotemporal gait parameters when the walkway system was compared to the reference systems. The findings from this analysis should provide confidence in using the walkway systems as reference systems in future studies to support the evaluation and validation of novel technologies deriving gait parameters.

## 1. Introduction

Wearable technologies that capture and analyze movement patterns for diagnostic or therapeutic purposes are a major locus of innovation in the United States. Wearable devices that derive spatiotemporal features of gait are being used as medical devices to support clinical decisions or as endpoints in clinical trials to support drug efficacy related to medical conditions or diseases that impact gait (e.g., Parkinson’s Disease, Multiple Sclerosis) [1,2,3,4,5,6,7,8]. When new technologies or methods deriving gait parameters are introduced (e.g., the use of inertial measurement units or sensorized insoles to characterize gait in remote settings), a measurement validation is performed to show evidence that the new method is reliable and valid and assesses what it purports to assess [9]. 

Ground reaction force plates and optoelectric motion capture systems are typically regarded as the “gold standard” or reference for the derivation of spatiotemporal gait parameters, as these systems have a large body of literature supporting their reliability and validity in capturing these parameters. Traditional stopwatch and step count approaches are still used in clinical practice and have been considered standard approaches for the calculation of some spatiotemporal measures. However, these traditional methods are subjective, and access to and the use of the force plate/motion capture systems may be difficult given the space requirements, set-up time, and/or constraints of a lab environment. As such, many researchers are moving toward the use of pressure-sensing walkways as the reference system with which to perform measurement validations [10,11,12,13,14,15,16,17,18,19,20]. The increasing interest in applying these types of systems to assess gait in a variety of clinical populations is mostly due to the quick set-up time, ease of use, and semi-automated processing of footfalls to extract hundreds of gait parameters. 

Understanding the importance of characterizing pressure-sensing walkaway technology for use in the derivation of spatiotemporal gait parameters, there have been several studies reporting on the reliability of the technology [21,22,23,24,25,26,27,28,29]. Reliability is an important measurement property to assess as it ensures the acquired data are representative of an individual’s actual performance and are stable over time [30]. Given the variable conditions under which many of these studies report reliability, a meta-analysis was performed in a recent systematic review to assess the test–retest reliability of specific gait parameters captured using different pressure-sensing walkways (i.e., gait speed, cadence, stride length, stride time, double support time, base of support) [30]. The results of that review suggest that, despite different populations and testing protocols used in the included studies, the test–retest reliability of the examined gait parameters was acceptable under single and cognitive dual-task conditions [30].

Another important measurement property to assess is concurrent validity. Concurrent validity can be defined as how well a measure agrees with a known criterion or established reference and generally requires that two measures be taken simultaneously. There are several studies reporting on the concurrent validity of these pressure-sensing walkways [31,32,33,34,35,36,37,38,39,40,41,42,43,44] but variable experimental protocols make it difficult to determine the overarching evidence supporting concurrent validity. Similar to the review conducted for test–retest reliability, the research and regulatory communities would benefit from a systematic review and meta-analysis of the existing literature examining the output of pressure-sensing walkways compared to established reference systems. 

The authors are not aware of a systematic review of studies conducted to assess the concurrent validity of pressure-sensing walkway technologies. The results of such an analysis could establish the body of evidence needed to confidently use these systems as reference systems when validating novel tools or measures. Therefore, the goal of this systematic review and meta-analysis is to assess the evidence of concurrent validity for pressure-sensing walkways. The review compared information related to the clinical population studied, the number of subject samples, the type of reference system used, and spatiotemporal gait measures to guide researchers and clinicians in the use and interpretation of data captured using the aforementioned systems. 

## 2. Methods

This review and analysis follow the Preferred Reporting Items for Systemic Reviews and Meta-analysis (PRISMA) statement. 

### 2.1. Literature Search Strategy and Selection Process

A systematic literature review was conducted to identify references related to gait analysis using pressure walkways published between 1980 and September 2023. The scope of the search was limited to Medline/PubMed, Embase, and Cumulative Index to Nursing and Allied Health Literature (CINAHL) databases. The following search terms were used in all databases: “gait” AND “human” AND (pressure walkway” OR “GAITRite” OR “Zeno walkway” OR “Tekscan”). Given the limited number of pressure-sensing walkways currently available on the market, the websites of each known pressure walkway vendor (i.e., GAITRite^TM^ (Franklin, NJ, USA), ProtoKinetics^TM^ (Havertown, PA, USA), and Tekscan^TM^ (Norwood, MA, USA)) were also reviewed for additional references. One reviewer conducted the literature search and collated all the studies (O.S.). Two reviewers (K.K. and O.S.) independently screened the article titles and abstracts to determine eligibility. A list of articles to consider from each independent reviewer was generated and discussed until a consensus on the articles to include in the systematic review and meta-analysis was reached. The quality of each study included in the review and analysis was also assessed using study quality assessment tools from the NIH National Heart, Blood, and Lung Institute. These tools generally consist of 14-question assessments for various types of studies. The possible assessment output for each study was either good, fair, or poor. Any studies with a quality assessment of poor were excluded from the meta-analysis. 

### 2.2. Selection Criteria

Articles were considered for inclusion if (1) a pressure-sensing walkway was used in the study, (2) spatiotemporal parameters of gait were calculated or the center of pressure during gait was assessed, (3) the output of the walkway systems was compared to an established reference system (optoelectric motion capture, force plates, or other reference systems) and not to another outcome measure (performance or patient-reported), (4) studies included a sample of at least 10 subjects (healthy or clinical population), and (5) studies were written in English. The included studies were limited to those written in the English language due to a lack of resources to accurately translate non-English studies. Publications were excluded from this review if (1) the pressure walkway was used as the reference system or (2) the article was not available in its full text. For the meta-analysis, the Bland–Altman studies reporting the mean and standard deviation of the differences between the pressure walkway and reference system (or if these two statistics can be derived from the reported data) were included.

### 2.3. Data Collection Process

For the systematic review, the following data were extracted from each eligible study: patient population tested, number of patient samples, type of pressure walkway system, type of reference system, length of walkway, task description, and gait parameters evaluated in the study. For the meta-analysis, two reviewers (O.S. and K.K.) extracted the mean and standard deviation of each spatiotemporal parameter for the pressure walkway and reference system, as well as the bias, upper and lower limits of agreement, and intra-class correlation coefficient (if available) for each included study. 

### 2.4. Statistical Analysis

The meta-analysis was performed using the R programming language, version 4.1.3. Among the included Bland–Altman studies, the agreement between the pressure walkway system and the reference systems was reported differently. For each metric, if the bias (i.e., mean difference) and standard deviation (i.e., the standard deviation of the difference), or equivalently the Limits of Agreement (LoAs), are reported, the results were used for the meta-analysis following the framework provided by Tipton and Shuster (2017) [45,46]. For some studies, the bias and standard deviations (or LoAs) were computed if the group mean and interclass correlation coefficients were reported. The plug-in-estimator by Tipton and Shuster (2017) [45,46] was used to estimate both the lower and upper bounds of the overall LoAs, which provides an estimate of the 95% confidence interval for the pooled LoAs. 

## 3. Results

**Figure 1:**
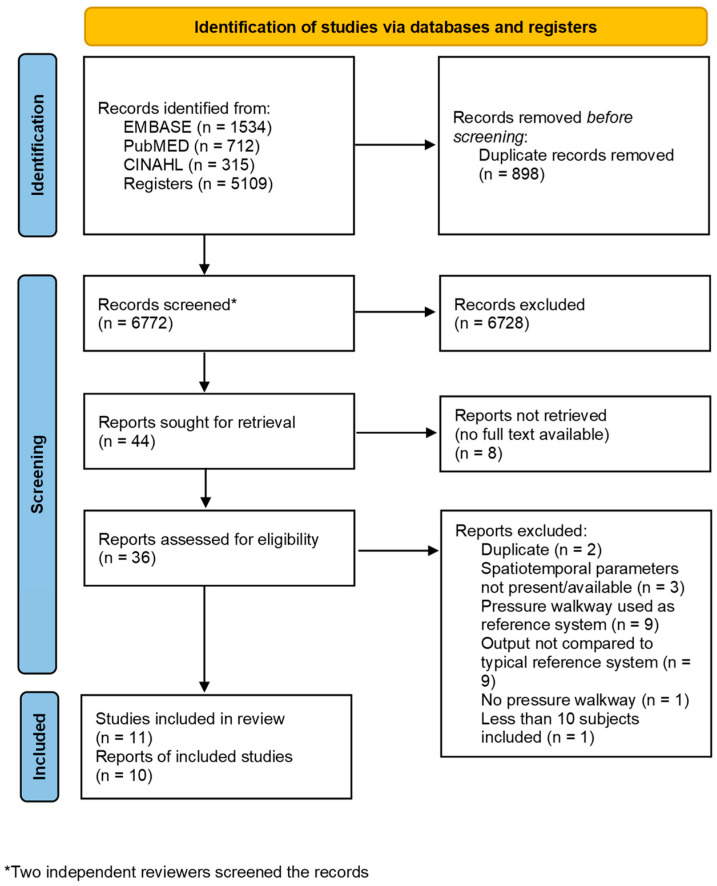
PRISMA flow diagram of identification, screening, and inclusion of articles for meta-analysis.

Based on these search terms, 7670 articles were identified. After removing duplicate articles, there were 6772 remaining articles. All articles identified as possibly eligible by each independent reviewer were sought for a full-text retrieval. Of the 44 articles that were assessed for eligibility, 11 were included in the systematic review. Most of the articles that were excluded during this full-text review were due to the walkway system being used as the reference system or due to the output of the walkway system being compared to an atypical reference (e.g., a patient-reported outcome). One article was excluded from the meta-analysis because the mean difference and standard deviation of the difference could not be derived from the data provided (Figure 1).

### 3.1. Characteristics of the Included Studies

Eleven studies were included in the systematic review. The table below (Table 1) provides details on each study including the study population tested, the number of participants, the type of walkway system used, the reference system used to establish concurrent validity, and the quality rating of the study according to NIH quality assessment tools. 

The most common patient population included in the studies was healthy controls followed by individuals with stroke. There were two studies focusing on the concurrent validity of the walkway in Parkinson’s Disease and cerebellar ataxia, respectively. All studies used the GaitRite walkway mat, but the lengths of the walkways used varied from 4.3 m to 8.4 m. Motion capture was more commonly used as the reference system, with a handful of studies using either traditional stopwatch–step count methods or footswitches to determine spatial and temporal gait parameters.

All studies required participants to walk across the walkway at a self-selected pace on a flat surface. Methods described within each paper indicate study administrators would tell participants to walk at a comfortable or at their preferred walking pace. For some studies, the same participants were also asked to walk at different speeds [36,38,41,43] in order to assess the validity of the walkway in capturing spatiotemporal gait parameters as a function of slow, normal, and fast walking speeds. 

### 3.2. Gait Parameters

There were 25 spatial and temporal gait parameters extracted from the 11 studies. The necessary data to perform the meta-analysis could not be extracted from one study (Schmitz-Hubsch et al., 2016 [31]), so that study was removed from the meta-analysis. From the remaining 10 studies, data for the following gait parameters were extracted: gait speed (m/s), cadence (steps/min), step length (cm), step time (s), stance time (s), swing time (s), stride length (m), stride time (s), stride velocity (cm/s), stance percentage (%), swing percentage (%), single support percentage (%), double support percentage (%), stance phase duration for left (s), stance phase duration for right (s), swing phase duration for left (s), swing phase duration for right (s), single support percentage left (%), single support percentage right (%), double support percentage left (%), double support percentage right (%), step length left (cm), step length right (cm), step time left (s), and step time right (s). 

Table 2 displays the estimated population bias and LoA for all gait parameters, as well as the number of distinct populations (N) included in the meta-analysis.

**Figure 2:**
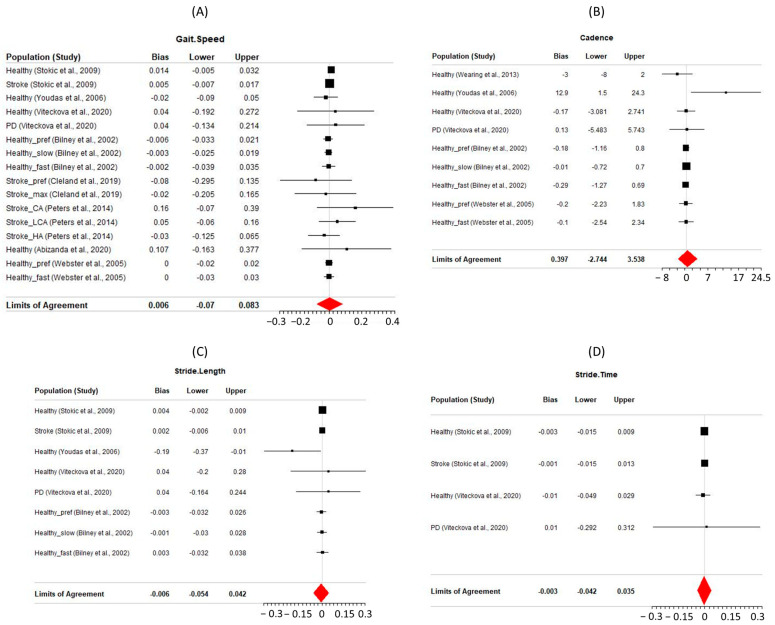

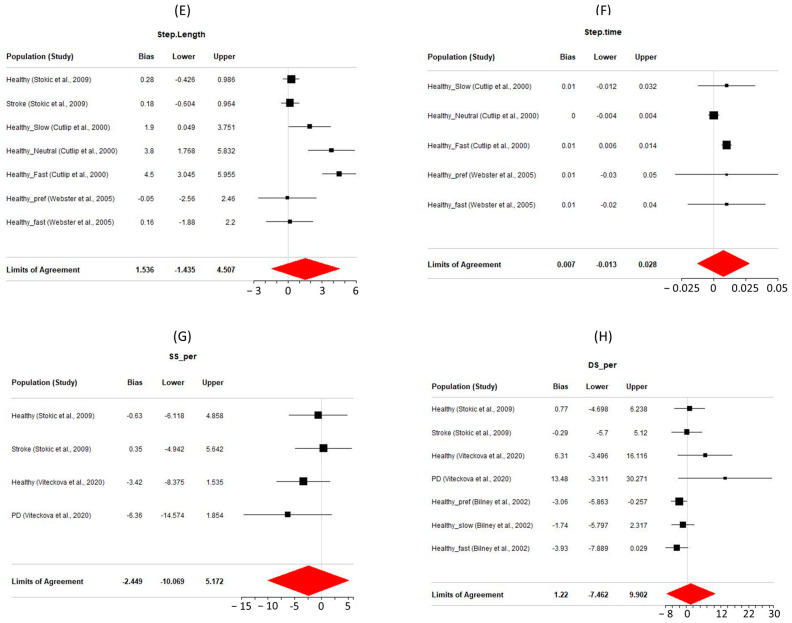
Forest plots of eight gait parameters: (**A**) gait speed (m/s), (**B**) cadence (steps/min), (**C**) stride Length (m), (**D**) stride Time (s), (**E**) step length (cm), (**F**) step Time (s), (**G**) single support (%), (**H**) double support (%).

Forest plots displaying the reported bias and LoA from each population within a study for gait parameters with four or more study populations are shown in Figure 2; these eight gait parameters (gait speed, cadence, step length, step time, stride length, stride time, single support percentage, and double support percentage) will be the focus of the results. The solid vertical line in each forest plot represents the line of no bias at x = 0; the red quadrilateral shows the estimated population bias at the center vertices and pooled upper and lower bounds of LoAs at the right and left vertices of the quadrilateral, respectively.

For all gait parameters of focus, the analysis results indicate reasonable agreement among different studies. Gait speed (plot A of Figure 2) included 16 different populations across eight studies and showed the walkway system only slightly overestimated the reference systems by an estimated bias of 0.006 m/s with LoAs between −0.07 and 0.083 m/s. Similar results were seen for stride length, stride time, and step time; the walkway system only slightly underestimated stride length (estimated bias = −0.006 m) and stride time (estimated population bias = −0.003 s) and slightly overestimated step time (estimated bias = 0.007 s). Larger confidence intervals were calculated for the single support and double support percentage of gait cycle parameters, but these meta-analysis results still indicated that the walkway system was not significantly different from the reference systems used to capture these same parameters.

## 4. Discussion

The evidence supporting the concurrent validity of pressure-sensing walkways used to determine spatiotemporal gait parameters was systematically collected and analyzed in this study. Results showed there was not a significant heterogeneity in terms of nearly all spatiotemporal gait parameters when the walkway system was compared to the reference systems.

For most gait parameters, the meta-analysis results showed the walkway system slightly overestimated the gait parameters, but the biases were not significantly different from zero. The conclusion from this analysis should provide confidence in using the walkway systems as reference systems in future studies to support the evaluation of novel technologies deriving gait parameters. Stride velocity was the only gait parameter for which the bounds of the LoAs did not include zero, indicating there was a significant overestimation of stride velocity when using the walkway system. All data from this conclusion came from a single study reporting on the difference between the walkway system and a video-based motion capture system while healthy participants walked on the mat at slow, neutral, and fast paces. Additional investigation into the validity of the walkway in capturing this gait parameter may be warranted.

Although there exist a handful of walkway system technologies capable of collecting pressure data as people walk across the mat, the only walkway system that was included in the eligible studies was the GaitRite^®^ (CIR Systems Inc., Havertown, PA, USA). The Zeno™ walkway (Protokinetics LLC, Haverton, PA, USA) was commonly referenced in the reviewed studies but was excluded mainly due to the fact the walkway system was being used as the reference system. Some studies reporting on the reliability and validity of the Zeno™ walkway in the healthy and older adult population determined the concurrent validity compared to the GaitRite^®^ system to be moderate to excellent [39] and the test–retest reliability and concurrent validity when measuring the center of pressure compared to force plates to be acceptable [21]. Another study compared the processing software between the two systems using the same footsteps collected from older adults and concluded there was minimal difference in the derivation of most gait parameters, suggesting the two systems and software may be used interchangeably [47]. The Tekscan Strideway (Tekscan, South Boston, MA, USA) is another system that has been used to assess spatiotemporal gait parameters in various populations, but most studies using this system were not focused on validation [48,49,50]. One conference paper reported on the concurrent validity of the Tekscan Strideway system to capture kinetic parameters compared to a force plate system and concluded vertical force measurements were comparable between the two systems [51]. Even with this evidence supporting the validity of the Zeno™ and Tekscan Strideway walkways and the many papers using these systems for the evaluation of gait, confidence in the use of these systems would be bolstered with a direct evaluation of concurrent validity as compared to a more commonly used reference system.

Although there were limited studies included in the review and meta-analysis, it is interesting to note that stride width and double support time were not included in any reviewed study. Stride width is defined as the horizontal distance between heels during double stance and is commonly referred to as the ‘base of support’ or ‘walking base’ [52]. The stride width for healthy adults is typically in the range of 50–130 mm but may be increased or decreased in pathological gait [53]. Similarly, double support time is commonly included as a temporal gait parameter to assess the impact of interventions on gait or evaluate disease progression [54,55,56]. Despite the common usage of these gait parameters, no studies were identified that report on the validity of using the walkway systems for capturing these parameters. 

The tasks performed by participants within each study were generally the same; participants were asked to walk across the walkway systems while data were being recorded simultaneously from the walkway system and a reference system. However, all studies were performed on a flat surface as participants walked in a straight line, limiting our understanding of the concurrent validity of these walkway systems in more real-world contexts. Daily ambulation in a real-world context requires a wider range of gait activities than straight, level walking, making the investigation of different walking motions during the use of walkway systems vital in gait parameter calculation. Individuals encounter obstacles in the form of curbs, stairs, cones, or other people, which forces them to deviate from the typical straight-line walking recorded during laboratory testing. Studies have shown that more than 30% of the walking time during community ambulation is spent along curved trajectories [57,58]. The evidence presented in this meta-analysis is a necessary step to accepting the walkway systems as adequate reference systems but further investigation into the concurrent validity of these systems with data from individuals performing more complex ambulation patterns is needed.

While the tasks performed were generally the same, there was variability in the selection of data for analysis. All studies acknowledged the importance of using data from steady-state gait when determining the concurrent validity of the walkway systems, but there was variability in the methodology to reduce the impact of acceleration and deceleration on the calculation of gait parameters. There were two approaches taken to limit the data used in the concurrent validity analysis in the studies: the use of “acceleration/deceleration zones” or the removal of steps at the beginning and end of a walking trial. Of the 11 studies included in the meta-analysis, 9 studies incorporated an acceleration/deceleration zone to achieve steady-state gait during recording on the walkway. The remaining two studies removed the “first and last stride onto the mat” [32] or the first and last steps on the mat [42]. The size of the acceleration/deceleration zone at the beginning and end of the walkway was different across the studies. One study instructed participants to begin “walking a few steps before the walkway and [continue] for a few steps after coming off the walkway” [33], while others required participants to start and stop 1 m [39], 2 m [34,35,38,40,43], 3 m [36], or 4 m [41] away from the walkway. Prior research has shown that steady-state gait is typically achieved within the first four steps for younger adults [59,60] and within three steps for healthy adults on a sloped incline [61]. Elderly persons tend to have more variability in specific gait parameters within the first four steps [60] but one study concluded that an acceleration/deceleration zone of approximately 2.5 m should suffice for the frail elderly population [62]. Given that nearly all included studies incorporated approaches that remove the first couple of meters of data from the analysis, errors in the spatiotemporal gait parameter derivation should be limited.

A discussion on the concurrent validity evidence for the gait parameter stride velocity is warranted given recent approvals by the European Medicines Agency for a digitally derived stride velocity 95th percentile metric for use in Duchenne Muscular Dystrophy trials [63]. The reports for concurrent validity for stride velocity in the current meta-analysis show the walkway overestimates stride velocity, with an estimated population bias of 10.63 cm/s. For a given clinical population, this bias may be larger than the expected difference in stride velocity over the course of a disease or after medical intervention, making it difficult to discern the impact of the disease or intervention [64]. However, these data points all came from a single study, limiting the generalizability of the results. Additional concurrent validation should be conducted for this gait parameter to determine if walkway systems are an appropriate reference system to use in clinics or for wearable sensor validation. 

It is important to note in the assessment of the methodological quality, that while all included studies were considered adequate, the results should also be interpreted in light of some limitations. The main strength lay in the rigorous systematic process and methodological quality assessment achieved through the use of the PRISMA checklists, a widely validated tool used to report review findings and to examine the risk of bias in reliability studies. Another strength was the relatively comprehensive literature search without the inclusion of search filters that could potentially lead to bias. However, we did not consult all electronic databases available and excluded text that was not written in English. Thus, there is a slight possibility of missing some studies that could be relevant to the present systematic review. In addition, this review did not consider studies enrolling pediatric populations (age < 18 years old). Thus, it is unclear whether the results from this systematic review are generalizable to studies wishing to assess the concurrent validity of pressure-sensing walkways used to collect data from pediatric populations. Future studies should include and review the reliability of gait parameters in this population sample, both in healthy and pathological conditions. In addition, we did not include any other exploratory subgroup analysis to investigate how possible confounding factors influence reliability results for each subset of individuals, due to the limited number of available studies that limited possible comparisons. The systematic review also emphasizes the lack of standardization in gait analysis protocols and should be a focus of future work to ensure consistency and comparability across studies. Finally, all of the included studies investigated used the GAITRite^®^ (CIR Systems Inc.) walkway as the reference walkway, and limited information is available for the other pressure-sensitive walkways. Consequently, this review did not robustly detect which pressure-sensitive walkway differences could influence the concurrent validity results for the different clinical populations. In conclusion, future studies should examine how possible confounding factors could influence the reliability results of gait parameters captured using pressure-sensitive walkways, as well as investigate their intra- and inter-rater reliability with other gait analysis instruments and clinical tests in different clinical populations. 

## 5. Conclusions

This systematic review of the evidence supporting the concurrent validity of pressure-sensing walkways showed the variety of populations in which these systems are being used and the various lengths of walkways used and revealed the limited data available from different manufacturers of this technology. Even with different populations assessed and variable approaches to analyzing steady-state gait footfalls to derive spatiotemporal gait parameters, the meta-analysis revealed that the walkway systems have reasonably good agreement with the reference systems for nearly all gait parameters included. The findings from this analysis should provide confidence in using the walkway systems as a reference system in future studies to support the evaluation of novel technologies deriving gait parameters.

## Figures and Tables

**Table 1:** Extracted information from studies included in the systematic review.

Reference	Study Population	# Participant	Walkway System	Reference System	Study Quality
Schmitz-Hubsch et al., 2016 [31]	Healthy	9	GaitRite	Motion capture system-IMU	Good
Schmitz-Hubsch et al., 2016 [31]	Cerebellar Ataxia	8	GaitRite	Motion capture system-IMU	Good
Wearing et al., 2013 [32]	Healthy	39	GaitRite	Footswitches	Good
Stokic et al., 2009 [33]	Healthy	52	GaitRite	Motion capture system–optoelectric	Good
Stokic et al., 2009 [33]	Stroke	20	GaitRite	Motion capture system–optoelectric	Good
Youdas et al., 2006 [34]	Healthy	40	GaitRite	Stopwatch–Count	Fair
Viteckova et al., 2020 [35]	Healthy	40	GaitRite	Motion capture system–IMU	Fair
Viteckova et al., 2020 [35]	PD	24	GaitRite	Motion capture system–IMU	Fair
Bilney et al., 2002 [36] *	Healthy_pref	25	GaitRite	Footswitches	Good
Bilney et al., 2002 [36] *	Healthy_slow	25	GaitRite	Footswitches	Good
Bilney et al., 2002 [36] *	Healthy_fast	25	GaitRite	Footswitches	Good
Cleland et al., 2019 [38] *	Stroke_pref	77	GaitRite	Stopwatch–Count	Fair
Cleland et al., 2019 [38] *	Stroke_max	77	GaitRite	Stopwatch–Count	Fair
Peters et al., 2014 [40] *	Stroke_CA	26	GaitRite	Stopwatch–Count	Fair
Peters et al., 2014 [40] *	Stroke_LCA	24	GaitRite	Stopwatch–Count	Fair
Peters et al., 2014 [40] *	Stroke_HA	12	GaitRite	Stopwatch–Count	Fair
Cutlip et al., 2000 [41] *	Healthy_Slow	10	GaitRite	Motion capture system–video	Good
Cutlip et al., 2000 [41] *	Healthy_Neutral	10	GaitRite	Motion capture system–video	Good
Cutlip et al., 2000 [41] *	Healthy_Fast	10	GaitRite	Motion capture system–video	Good
Abizanda et al., 2020 [42]	Healthy	90	GaitRite	Motion capture system–video	Fair
Webster et al., 2005 [43] *	Healthy_pref	10	GaitRite	Motion capture system–optoelectric	Good
Webster et al., 2005 [43] *	Healthy_fast	10	GaitRite	Motion capture system–optoelectric	Good

* Additional descriptors of gait speed are provided in the patient population section if studies explored the impact of gait speed on validation—‘pref’ is preferred speed, ‘fast’ is fast speed, and ‘slow’ is slow speed. All other studies with no additional descriptors used the participant’s preferred speed during the study. The Peters et al. [40] study explored validation for stroke patients who were classified as community ambulators (CAs), low community ambulators (LCAs), and high ambulators (HAs).

**Table 2:** Summary of meta-analysis for all gait parameters.

Gait Parameter	N	Estimated Population Bias	Estimated Lower LoA	Estimated Upper LoA
Gait speed (m/s)	16	0.006	−0.07	0.083
Cadence (steps/min)	9	0.397	−2.744	3.538
Stride length (m)	8	−0.006	−0.054	0.042
Stride time (s)	4	−0.003	−0.042	0.035
Step length (cm)	7	1.536	−1.435	4.507
Step time (s)	5	0.007	−0.013	0.028
Single Support (%)	4	−2.449	−10.069	5.172
Double Support (%)	7	1.22	−7.462	9.902
Stance time (s)	3	−0.007	−0.035	0.021
Swing time (s)	3	0.003	−0.024	0.03
Stride velocity (cm/s)	3	10.63	4.549	16.71
Left step length (cm)	3	0.857	−2.213	3.926
Right step length (cm)	3	0.307	−2.457	3.071
Left step time (s)	2	0.004	−0.018	0.026
Right step time (s)	2	0	−0.037	0.038
Stance phase (%)	2	4.826	−3.062	12.714
Swing phase (%)	2	−4.816	−12.704	3.072
Left stance phase (s) *	1	−1	−2.2	0.02
Right stance phase (s) *	1	−1	−2.6	0.06
Left swing phase (s) *	1	1	−0.2	2.2
Right swing phase (s) *	1	1	−0.6	2.6
Left single support (%) *	1	1	−0.7	2.7
Right single support (%) *	1	1	−0.2	2.2
Left double support (%) *	1	−2	−4.6	0.6
Right double support (%) *	1	−2	−4.5	0.5

* For those parameters with N = 1, there is only one population and the bias and LoAs were directly reported in the referenced study without pooling.

## Data Availability

The raw data supporting the conclusions of this article will be made available by the authors on request.
